# Effect of osteopenia and osteoporosis on failure of first and second dental implants: a retrospective observational study

**DOI:** 10.1186/s40729-024-00556-9

**Published:** 2024-09-04

**Authors:** Nathalie Frumkin, Jennifer Ana Iden, Devorah Schwartz-Arad

**Affiliations:** Schwartz-Arad Surgical Center, 15 Habarzel St, Tel Aviv, Israel

**Keywords:** Endosseous dental implants, Implant survival, Implant failure, Osteoporosis, Osseointegration, Re-implantation

## Abstract

**Purpose:**

The present study evaluated osteopenia (OPN) and osteoporosis (OP) as risk factors for dental implant failure and repeat failure.

**Methods:**

We performed a retrospective study on over 100 randomly selected patients per analysis to determine the effect of health status, smoking status, sex, implant location and operative conditions on first and second (re-implantation) implant survival. Analyses were conducted first using chi-squared test, followed by multiple logistic regression for significant variables.

**Results:**

In the cohort examining the effect of myriad risk factors on second implant survival, it was found that OPN and OP greatly impacted implant survival, wherein patients with osteoporosis or osteopenia had significantly more implant failures (p = 0.0353). Sex and operative conditions had no effect on implant survival, while implant location showed a notable effect wherein significantly more failures occurred in the maxilla vs mandible (p = 0.0299). Upon finding that OPN and OP have a significant effect on second implant survival, we conducted an additional study focusing on the impact of health status. Based on the multiple logistical regression analysis, we found that OPN and OP are the most significant factor in first implant survival (p = 0.0065), followed by diabetes (p = 0.0297). Importantly, it was observed that early implant failure is also significantly correlated with osteoporosis (p = 0.0044).

**Conclusion:**

We show here a marked relationship in which the risk of first and second implant failure are significantly higher in patients with osteoporosis and osteopenia.

**Supplementary Information:**

The online version contains supplementary material available at 10.1186/s40729-024-00556-9.

## Background

Dental implants are a predictable and reliable treatment modality for edentulous patients and usually provide favorable, highly successful results [1]. However, implant failures still occur for a variety of reasons [2, 3]. As a result, the risk factors for dental implant failure are now a commonly addressed subject in current dental research [4, 5].

The outcome for implant restoration has been thought to be influenced by a number of factors, including implant design (length, shape, or surface texture), patient-related medical risk factors (systemic diseases or habits, such as smoking), and surgical complications [6, 7]. With the significant improvements in surgical and material technology, patient-related disorders are receiving more attention as potential risk factors for dental implant failure and repeated failure.

Replacement implants have been shown to have a lower survival rate than initial implants [8]. This can be attributed to several factors, including inadequate osseointegration, which may result from poor bone quality or quantity, insufficient initial stability, or premature loading of the implant [9]. Recurrence of peri-implantitis, an inflammatory condition affecting the tissues around the implant caused by bacterial infection, is also a major cause of implant failure upon retreatment [10].

It has previously been indicated that local and systemic risk factors can result in higher failure rates [11]. Medical disorders like diabetes, osteoporosis (OP), obesity, and medication use can all impede bone repair around dental implants [12]. Diabetes mellitus, a chronic disease resulting in hyperglycemia which causes multifarious symptoms, is hypothesized to be a relative contraindication for implant surgery, yet is still controversially discussed [13–15]. A potential cause for the discrepancy is due to the number of patients suffering from diabetes is increasing, so there are more diabetic patients demanding implant procedures [16, 17]. OP has similar controversial representations in the literature with regard to implant survival [12, 18–20].

OP and osteopenia (OPN) are both conditions characterized by a decrease in bone density, leading to an increased risk of fractures; osteopenia is a milder form of bone loss compared to osteoporosis, serving as a precursor to the more severe condition [21]. While both conditions reflect reduced bone density, osteoporosis is distinguished by a bone density that is significantly lower [22, 23].

OP is the most common metabolic bone disease, presenting with uncoupled bone resorption, which results in reduced bone mass, impaired microarchitecture, and structural deterioration—commonly referred to as fragility fractures [23–25]. These factors increase the risk of fracture with little or no external force. Both men and women can develop OP, and one of the main causes of primary OP in women is menopause [26]. As such, the majority of women who are in their sixth and seventh decades of life are affected by OP, with the lower age limit of diagnosis around 50 years [27, 28].

Patients suffering from OP undergoing bone resorption inhibitor therapy, such as bisphosphonates and denosumab, show an increased risk of developing osteonecrosis of the jaw after an oral surgery [29–32]. As in most clinics, the treatment is performed according to the type of bone resorption inhibitor and the timing of intravenous injections in addition to a serum test for bone turnover markers [33, 34].

The association between OP and dental implant failure is still under debate. While some reports have found no association between the disease and implant failure, anecdotal and observational experience in the clinic dictates otherwise [12, 18–20]. Due to the decrease of bone density in OP and OPN patients, we hypothesized that these conditions would increase the risk of dental implant failure and repeated failure. Therefore, we conducted a retrospective study using randomly selected patients to establish the relationship between OP and OPN incidence, as well as other prevalent diseases, and implant survival in first and second implant attempts.

## Methods

All patients included in the study received treatment by DSA. While the majority of patients received treatment solely by DSA, a small portion of patients received treatment intermittently by other dental practitioners.

### Study population selection

#### Sample size determination and random sample selection

We determined the sample size using the formula that considers a finite population, wherein margin of error (ε) is 5%, z is the z score, N is the population size, and p̂ is the population proportion.

$$n\prime \, = \,\frac{n}{{1 + \frac{{z^{2} \times \hat{p}\left( {1 - \hat{p}} \right)}}{{{\upvarepsilon }^{{2}} {\text{N}}}}}}$$

For the 2nd implant survival study, the population proportion is 7%, as this is the failure rate of implants seen in this clinic and in the general population, and N is 6000 (total number of patients seen at the clinic) [35, 36]. The n found is 86, which was increased by 20% to 103 to accommodate for missing information after random selection. Similarly, for the first implant survival study, the population proportion is 9.5%, as this the occurrence of dental implant failures in this clinic in patients over 50 years old and is within the range of reported failure rates for this age group [37, 38]. The n found is 130, which was increased by 20% to be 156 in order to accommodate for missing information after random selection. The resulting number of patients included in the analysis of failures were 152 (1st failure analysis) and 94 (2nd failure analysis).

Lists of patients with at least one implant failure (2nd implant survival study) and at least one implant (1st implant survival study) performed by DSA during the designated time frames were generated using the electronic health record software system (Rapid-Image) and exported to Microsoft Excel, wherein random selection from a list without duplicates was performed.

*Second implant survival*: 112 randomly selected patients with at least one implant failure between 1990 and 2020 were included in this cohort. All relevant information was recorded for each patient. All patients included in the analysis of second implant failure received a second implant after the first implant failed: 94 out of 112 patients received a second implant. Patients lacking a particular documentation were excluded from the analysis for that specific parameter.

*First implant survival*: 152 patients randomly selected patients over 50 years of age who received implants between 1994 and 2018 were included in this cohort. All relevant information was recorded for each patient. The purpose of this study was to determine the effect of health-related disorders on first implant failures. Therefore, an additional exclusion criterion was considered on a case-by-case basis wherein patients lacking a complete medical history or that did not completely or clearly fill in the medical history survey were excluded from the study. On the health questionnaire provided by this clinic, there is a specific inquiry for OP and OPN. These diagnoses were confirmed with health reports from primary care or treating physicians. Many patients were diagnosed with OPN prior to receipt of implants, and as their treatment in the clinic progressed, they were diagnosed with OP, although it is important to note we were not always informed in a timely manner. Because OPN patients also have lower bone density (although not as low as OP patients), we classified all patients with OPN or OP as a “positive” diagnosis in a binary classification system.

*Implants*: The implants included in this study were endosteal titanium implants, the majority of which were tapered-body with an internal hex connection (Tapered-Screw Vent^®^ by ZimVie Biomet, Legacy^™^ and Legacy3™ by Implant Direct and spiral implants Spiral^®^ and Spiral Flare Bevel by Alpha-Bio Tec), accounting for 87% of implants. The remaining 13% of implants were spline implants with external connection (Spline^®^ Implant System, Zimvie Biomet) and straight-body implants with an internal hex connection (Core-Vent, Paragon^®^), accounting for 5.5 and 7.5% of implants, respectively. The lengths of implants ranged between 11.5 and 16 mm.

### Statistical analysis

To assess the risk factors for 2nd implant survival, we performed multiple χ^2^ tests and Fisher’s exact test in cases where n < 6, comparing the number of patients with at least one implant failure and the number of patients with all implants surviving.

For the 1st implant survival study, we again performed multiple χ^2^ tests as above. Based on the variables significant in the χ^2^ test, we investigated the associations of those variables with the corresponding implant survival or failure outcome by means of multiple logistic regression analysis. The 95% confidence interval (CI) was applied to test the significance of each regression coefficient in the regression model. If the 95% CI for a coefficient did not cover zero, there was less than 5% chance (*p* < 0.05) that the coefficient was zero, which was considered significant. All analyses were performed using Graph Pad Prism.

### Dummy variables

All clinical indications were defined as 0 (no) or 1 (yes), *i.e.* implant failures were defined as 1 and implant survival was defined as 0. Analysis was also completed for the number of failures in a multiple regression model (data not shown).

### Ethics committee

This is a retrospective, observational archival study wherein no information with any identification markers is revealed, and was conducted in accordance with the Helsinki Declaration of 1975, as revised in 2013. All data used in this study is observational data routinely collected. All patients consented to the possibility of using their information anonymously for research. The data was extracted and analyzed in the same clinic as was collected, so no additional permissions were necessary. This work is compliant with the STROBE checklist.

## Results

### *OPN and OP increase the risk of 2*^*nd*^* implant failure*

**Fig. 1:**
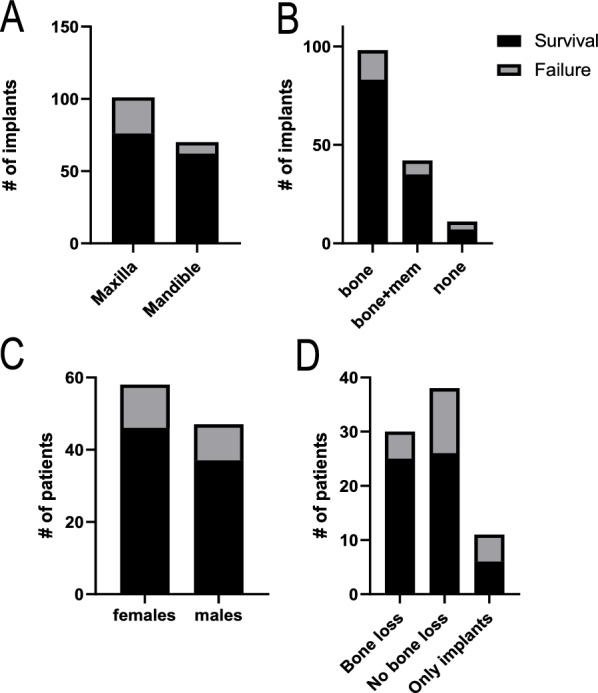
Distribution of survival and failure of second implants. **A** implant location **B** operative conditions of implantation including bone additives with or without a membrane (mem) or no additives (none) **C** sex **D** bone loss directly prior to implantation. Bone loss and sex were recorded per patient and implant location and operative conditions were recorded per implant

**Fig. 2:**
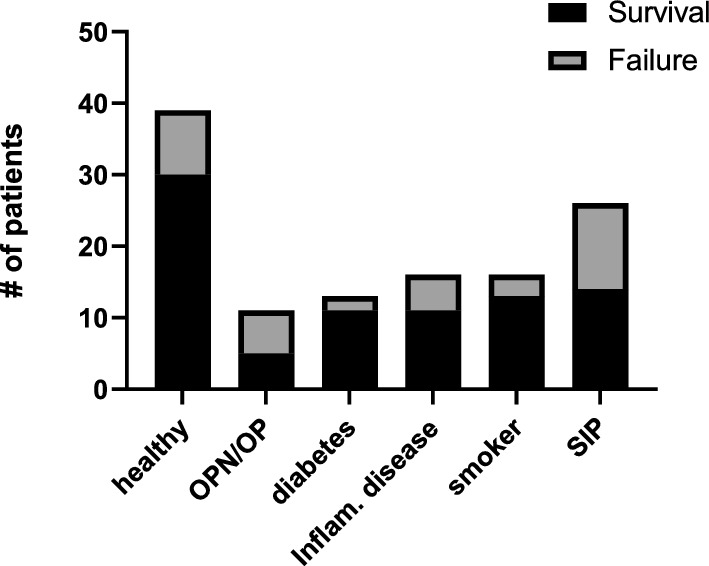
Distribution of health status in patients receiving a second implant. Includes overlap between patients with more than one clinical indication. *SIP* smoked in the past

The analysis of second implant survival involved randomly selecting 112 patients (described in Table 1) who had at least one implant failure (1st failure, Fig. 1). We assessed the risk of 2nd implant failure for the following putative factors: sex, pre-existing health status (including diabetes, OPN or OP, or other inflammatory disorders), smoking status, bone loss prior to implantation, implant location, and operative conditions (augmentation, immediate implant placement, etc) (Fig. 2). Analyses of implant location and operative conditions were performed per implant, while all other parameters were assessed per patient. Interestingly, we found that most parameters listed above did not significantly increase the risk of 2nd implant failure (Table 2), although implants in the maxilla presented a higher failure rate compared to the mandible (Table 2, p = 0.0299). Importantly, we found that patients with OPN or OP had a significantly higher probability of 2nd implant failure compared to healthy patients (p = 0.044) and patients without OPN or OP (p = 0.0353).

**Table 1:** Study population characteristics

Study	Number of patients	Number of implants	Age range (y)	Sex	Implant system
2^nd^ implant failure	94	951	40–84	43 males; 51 females	823 tapered-body +IHC; 55 spline +EC; 73 straight-body +IHC
1^st^ implant failure	152	1229	50–84	50 males; 102 females	1069 tapered-body +IHC; 64 spline +EC; 96 straight-body +IHC

*IHC* internal hex connection, *EC* external connection

**Table 2:** Chi-square test or Fisher’s exact test of risk factors for 2nd implant failure

Risk factor	χ^2^	*p* value
Sex	0.0054	0.9414
Implant location	4.713	0.0299*
Operative condition	3.0891	0.2134
Bone loss	1.988	0.1585
Smoker: compared to non-smoker	Fisher’s	>0.999
Smoker: compared to healthy patients	Fisher’s	>0.999
OPN/OP: compared to all non-OPN/OP	4.432	0.0353*
OPN/OP: compared to healthy patients	4.4046	0.044*
Diabetes: compared to non-diabetics	Fisher’s	0.7264
Diabetes: compared to healthy patients	Fisher’s	0.7087

*p < 0.05

### OPN and OP increase the risk of 1st implant failure and early failure

**Fig. 3:**
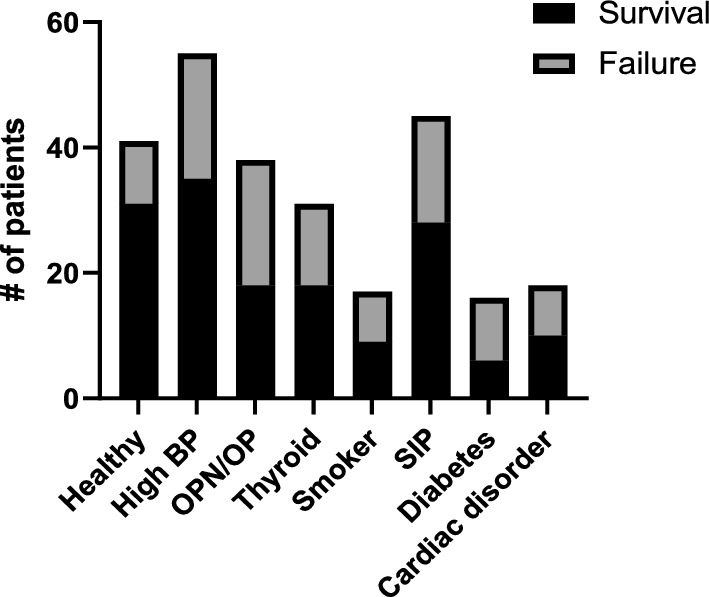
Distribution of health status in patients receiving at least one implant. Includes overlap between patients with more than one clinical indication. *BP* blood pressure, *SIP* smoked in the past

Due to the results seen in the analysis of second implant failures, we analyzed an additional cohort to determine if OPN or OP increases the risk of first implant failures as well. We randomly selected 146 patients over 50 years of age who received implants and assessed the impact of health status on implant failure: the influence of high blood pressure, OPN or OP, thyroid disorders, cardiac disorders, diabetes, and smoking status were assessed (Fig. 3). Corroboratively, we found that patients with OPN or OP have a significantly higher risk of 1st implant failure (Table 3, p = 0.0202, χ^2^) as well as patients with diabetes (Table 3, p = 0.0251, χ^2^). Notably, diabetes shows a significant effect on implant survival for first implants, but not on second/replacement implants (Table 2). We then performed a multiple logistic regression using the two variables that were significant in the univariate analysis, and found that OPN or OP patients have the highest risk (Table 3, p = 0.0065), followed by patients with diabetes (Table 3, p = 0.0297).

**Table 3:** Chi-square (univariate) and multiple logistic regression (multivariate) analysis of risk factors for 1st implant failure

	Univariate				Multivariate			
Risk factor	OR	95% CI	χ2	*p* value	OR (β)	95% CI	Wald	*p* value
High BP	0.958	0.4667–1.888	0.01468	0.9036				
OPN/OP	2.418	1.151–5.200	5.395	0.0202*	2.868	1.351–6.213	2.719	0.0065**
Thyroid	1.304	0.5737–2.905	0.413	0.5201				
Smoker	1.604	0.5670–4.248	0.8376	0.3601				
Diabetes	3.258	1.183–9.653	5.019	0.0251*	3.375	1.152–10.71	2.174	0.0297*
Cardiac	1.426	0.5190–3.649	0.49	0.4839				

*p < 0.05, **p < 0.01

**Fig. 4:**
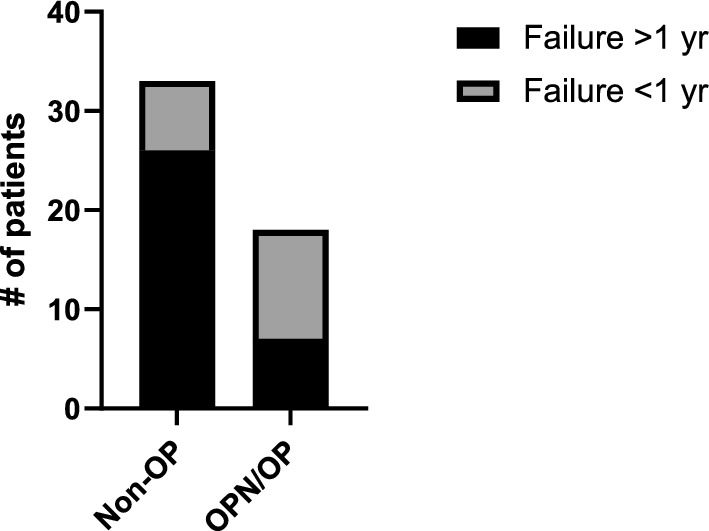
Distribution of osteopenia and osteoporosis in patients with late (>1 year) or early (<1 year) implant failure. Non-OP refers to all patients not diagnosed with osteopenia or osteoporosis. n > 6 per group, χ^2^, p = 0.0044

No relationship was determined regarding the time to implant failure and health status using a linear regression, as time to failure is variable between implants per patient. However, when comparing the number of patients with an early implant failure (<1 year after implantation) to late failures, we found that a significantly higher proportion of early implant failures occur in patients with OPN or OP (Fig. 4, p = 0.0044, χ^2^), indicating that these diseases potentially affects osseointegration, which has not previously been shown.

The analysis of 1st implant failure was not designed to accommodate an additional analysis of 2nd implant failure since all patients receiving implants were included, and 2nd failure rate is quite low in the general population meaning that an appropriate statistical evaluation cannot be done. However, it is important to note that out of eight 2nd failed implants in this cohort, four of them are from patients with osteoporosis or osteopenia.

## Discussion

Here, we show that pre-existing health conditions, specifically OPN or OP or diabetes, increases the risk of both first and second implant failure. As many previous studies on implant failure rates and risk factors were limited by a relatively small cohort, a single implant system, or a follow-up of less than 5 years [39], we designed this retrospective study to include > 100 patients for each failure (1st and 2nd) with substantial follow-up (maximum of 25 years) and myriad implant systems. The inclusion criteria for the 2nd implant failure did not have an age limit, while for 1st implant failure, we set the age limit at 50 in order to select for patients who are within the minimum age range to be diagnosed for osteopenia and osteoporosis. Out of 38 patients with OPN or OP in the cohort of first implant failure analysis, 34 were females. Because the data is heavily skewed towards females and this occurs in the general population as well, we did not assess females alone and included males in the final analysis. Additionally, we did not find any sex differences in risk of failure, confirming previous reports [40]. With regard to implant system, the majority of implants included in this study were titanium, tapered-body implants with an internal hex connection. While 13% of implants were spline implants or straight-body implants, the failure rates were similar among implant types (10–12% failure rate). Further studies designed specifically to determine if implant design is a confounding factor when considering OPN or OP patients should be conducted.

We have shown here for the first time that diabetes significantly affects 1st implant failure, but not repeat failures at the same site. It is possible that the initial implant procedure, despite failing, results in local alterations in the microenvironment of the implant, which contributes to the survival of consecutive implants in the same site. Although definitive evidence of a change in bone mass in patients with diabetes mellitus is not clear, it has been shown that bone quality does in fact decrease [41, 42]. Bone quality—characterized by bone turnover, microarchitecture, mineralization, microdamage and the composition of bone matrix—is potentially the main contributing factor for implant failure, as patients with osteoporosis suffer from a decrease in bone quality as well [43].

As prefaced above, the data reflects the general population. We initially found in our study on 2nd implant failures, OP significantly impacts the chance of implant survival. For the 2nd cohort of patients in the implant failures study, we selected for patients over 50 years of age, when the symptoms and diagnoses of OP begin [44]. Because of the many cases of early implant failures coupled with OP, we considered the impact of selecting for a population of older patients. We were able to demonstrate that our data is reflective of reported incidences and is not biased. Specifically, occurrences of OP only and OPN are 18.5% and 7.5% in our study (Supplementary Table 1). It has been reported that in adults over 50, OP occurs in about 15-20% and OPN occurs in about 4–20% of the population [23, 45]. Similarly, early implant failure is estimated to occur in 0.76–7.47% of cases [46]. We found 1.4% of implants failed in under 1 year (Supplementary Table 2). Implants have an average survival rate of 85–95% [7, 47]. Out of 1229 implants in this study of first implant failures, only 134 failed, showing comparable results (89% survival rate, Supplementary Table 2). The acceptable general failure rate strengthens our hypothesis that implant failure seen in the study is partly due to systematic conditions such as diabetes and OP, rather than common surgical complications. The second implant failure rate seen in the study is 20%. While this failure rate is higher than some modern reports, the documented range is substantial (4–29%), and the follow-up on patients therein was typically between 1 and 10 years [8]. This could indicate why the failure rate seen in this cohort was higher than average, as the follow-up ranges from 2 to 25 years, with a mean of 12 years.

In the literature, there are conflicting reports regarding the relationship between OP and implant failure [39, 48]. While many studies claim that OP is associated with implant failure, others claim there is no association. To our knowledge, most of these studies were conducted on a small sample size and meta-analyses/systematic reviews petition randomized clinical studies. Since osteoporosis occurs in the older population, coupled with its relatively low occurrence when factoring in people of all ages, we provide here a more definitive conclusion, although the mechanism by which osteoporosis influences implant failure or osseointegration has not yet been elucidated.

The Receptor Activator of Nuclear Factor κB-Ligand (RANKL)/osteoprotegerin (OPG) ratio is central to modulating bone healing and remodeling [49, 50]. This ratio is crucial to proper bone turnover, and because osteoporosis patients suffer from dysregulated RANKL/OPG, we speculate that this systemic deficiency could contribute to implant failure [51]. It is routine for clinicians to test osteoporotic patients for N-terminal propeptide of type I procollagen (PINP) and C-terminal telopeptide of type I collagen (CTX) for risk of bone necrosis prior to more complicated procedures [33]. Despite this caution, other factors are not assessed. Cross-talk among bone turnover and resorption markers could lead to an overlap wherein patients may still be in the osseointegration process with elevated alkaline phosphatase levels and depleted osteocalcin levels, leading to an increased risk of failure.

## Conclusions

Taken together, our results show that diabetes mellitus appears to impact only first implant failures and not repeat failures. Osteoporosis and osteopenia are associated with greater risk of first and repeat implant failure, as well as early implant failure, possibly due to lack of osseointegration.

## Supplementary Information

Supplementary Material 1.

## Data Availability

The datasets used and/or analysed during the current study are available from the corresponding author on reasonable request.
