# Supplementary material for: Effect of osteopenia and osteoporosis on failure of first and second dental implants: a retrospective observational study
Source: Int J Implant Dent. 2024 Sep 4;10:40. doi: 10.1186/s40729-024-00556-9 (PMC11374952; doi:10.1186/s40729-024-00556-9)
Supplement: Supplementary file 1 — Supplementary Material 1. [file 40729_2024_556_MOESM1_ESM.docx]

Supplementary material

**Supplementary Table 1. Occurrences of OP and OPN**

**Supplementary Table 2. Occurrences of implant failures**

|  | % of patients |
| --- | --- |
| with OP or OPN | 26 |
| with OPN only | 7.5 |
| with OP only | 18.5 |

|  |  | % of total implants |
| --- | --- | --- |
| # of implants | 1229 | X |
| # of failed implants | 134 | 10.90317 |
| # of early failures | 18 | 1.464605 |

**Supplementary Table 3. Implant failures in patients with OP or OPN**

|  | total | OP or OPN | non-OP/OPN* | Healthy** |
| --- | --- | --- | --- | --- |
| # of patients | 146 | 38 | 108 | 41 |
| # of patients with failed implant | 54 | 20 | 34 | 31 |
| # of patients with early failure (<1 year) | 16 | 9 | 7 | 2 |

*Non-OP/OPN patients are patients without OP and OPN only, includes other ailments.

** Healthy patients do not have ***any recorded*** ailments (OP, OPN, diabetes, arthritis, hypothyroidism, high blood pressure, etc).
